# Male Selenoprotein F-Like (SPF-L) Influences Female Reproduction and Population Growth in *Nilaparvata lugens* (Hemiptera: Delphacidae)

**DOI:** 10.3389/fphys.2019.01196

**Published:** 2019-09-25

**Authors:** LinQuan Ge, YongKai Zhou, HaoTian Gu, Qing Wu, Ze Zhou, Sui Zheng, David Stanley, QiSheng Song

**Affiliations:** ^1^School of Horticulture and Plant Protection, Yangzhou University, Yangzhou, China; ^2^Biological Control of Insects Research Laboratory, USDA/Agricultural Research Service, Columbia, MO, United States; ^3^Division of Plant Sciences, University of Missouri, Columbia, MO, United States

**Keywords:** *Nilaparvata lugens*, selenoprotein, seminal fluids, mating, fecundity

## Abstract

Selenoproteins serve in anti-oxidant and cellular redox functions in almost all organisms. A recent study characterized a selenoprotein F-like (SPF-L) in the brown plant hopper’s (BPH), *Nilaparvata lugens*, male accessory glands (MAGs), raised the question of whether the SPF-L is associated with female fecundity. In this study, *SPF-L* mRNA was found to be enriched in the internal reproductive organ (IRO) of virgin males, also expressed relatively stably in virgin males and females, and dietary dsSPF-L-treatments led to reduced MAG protein and Arginine content. Knockdown of *NlSPF-L* in unmated males did not influence juvenile hormone (JH) III and ecdysteroid titers, however, dsSPF-L-treated mated males had increased JH III titer, and reduced ecdysteroid titer compared to controls. After mating with dsSPF-L-treated males, female partners had reduced fat body and ovary soluble proteins and JH III tier and vitellogenin (Vg) mRNA levels, but no alterations in ecdysteroid titer, body weight or longevity. The experimental females had prolonged pre-oviposition periods and they laid fewer eggs, which suffered reduced hatching rates and population growth index (PGI). Such mating also led to impaired IRO development in males and females, which was confirmed by immunofluorescence staining. We infer that SPF-L affects reproductive success of males and their partners.

## Introduction

A great diversity of reproductive strategies have evolved in sexual organisms. Aside from sperm transfer, male seminal fluids have a role in post-copulatory reproductive success because they are involved in the fertilization processes and sexual selection in animals (Poinani, 2006). In some male insects, the seminal fluids, secreted mainly by accessory glands, contribute to the formation of a mating plug that prevents re-mating or sperm efflux. Seminal fluids protect sperm and alter female behavior and physiology. In particular, in some species, it leads to rejecting subsequent males and facilitating feeding, ovulation, egg production and longevity (Simmons, 2001; Ravi Ram and Wolfner, 2007; Avila et al., 2011; Clifton et al., 2014). Some proteins responsible for these events have been identified from the accessory glands of *Drosophila melanogaster* (Ravi Ram and Wolfner, 2007, 2009).

The brown planthopper (BPH), *Nilaparvata lugens* Stål (Hemiptera: Delphacidae) is a serious rice pest insect in Asia (Cheng, 2014), with a history of devastating outbreaks. Recent advances, such as new BPH-resistant rice varieties, new insecticides and integrated pest management programs help mitigate BPH damage. Nonetheless, BPHs poses real threats to rice cropping systems because some agricultural chemicals, such as the insecticide triazophs (TZP) and the antibiotic jinggangmycin (JGM), stimulate reproduction and population increases (Ge et al., 2010a; Wang et al., 2010). One of the molecular mechanisms of the increased reproduction is increasing male accessory gland protein (MAGP) content in males and increased MAGP transfer to females. The increased MAGP transfer leads to enhanced egg laying (Long et al., 2010) and almost life-long refractoriness to further insemination. Yu et al. (2016) used proteomic analyses to characterize the seminal fluid proteins composition in BPH male and some new examples were identified in male internal reproductive organs (IROs), and transferred to females via copulation, including a selenoprotein F-like (SPF-L), epidermal growth factor domain-containing proteins and a neuropeptide ion transport-like peptide. We suspect these and other transferred proteins are responsible for increased egg production and deposition in females.

Selenoproteins in human seminal fluid protect sperm during storage (Michaelis et al., 2014). Nevertheless, the roles of SPF-L in male BPH fertility and how SPF-L modulate female fecundity after copulation remain to be addressed. These proteins exist in almost all life forms. They feature the specialized amino acid selenocysteine in their reactive center, and mainly function as redox-enzymes (Stadtman, 1996). Selenium is essential for male fertility in mammals (Brown and Burk, 1973). Selenium deficiency leads to impaired sperm motility and flagellum loss (Brown and Burk, 1973), and diets lacking it result in shorter life spans (Martin-Romero et al., 2001).

Selenium is likely an essential micronutrient in all animals, which led to our hypothesis that male SPF-L influences male and female fitness, observed as an increase in offspring. Here, we report on the outcomes of experiments designed to test our hypothesis.

## Materials and Methods

### Rice Variety and Insects

The rice (*Oryza sativa* L.) cultivar, Ningjing 4 (japonica rice), is commonly planted in Jiangsu Province, China. After soaking and germination, the seeds were sown in outdoor standard cement tanks (height 60 cm, width 100 cm, and length 200 cm). The six-leaf seedlings were transplanted into 16 cm diameter plastic pots with four hills per pot and three plants per hill. All experiments were conducted with rice plants at the tillering stage.

Planthopper used in the experiments were obtained from a stock population maintained at the China National Rice Research Institute (CNRRI; Hangzhou, China). BHP were reared at Yangzhou University in an insect nursery consisting of rice plants covered with cages under natural conditions in cement tanks from April to October. The BPH were transferred into a greenhouse during winter. Prior to the experiments, the BPH colony was allowed to reproduce for two generations in an insectary under standard conditions, 26 ± 2°C, and photoperiod, 16L:8D, without insecticide application.

### dsRNA Synthesis

We designed gene-specific dsSPF-L primers and amplified a 272bp *SPF-L* (XM022339255) cDNA fragment using forward and reverse primers containing the T7 primer sequence at the 5′ ends (Table 1). The amplification program was 35 cycles of 94°C for 1 min, 94°C for 40 s, and 72°C for 1 min, with a final extension step of 72°C for 10 min. The sequence was verified by sequencing (Invitrogen, Shanghai, China). We used the *GFP* gene (ACY56286; provided by Chuan-Xi Zhang, Institute of Insect Sciences, Zhejiang University) as control dsRNA and amplified a 688 bp fragment using primers listed in Table 1. For *NlSPF-L* and the control GFP gene, we used the T_7_ RiboMAX^TM^ Express RNAi system (Promega, Sunnyvale, CA, United States) for dsRNA synthesis, following the Promega instructions.

**TABLE 1:** PCR primers used in this study.

**Primers**	**Sequence**	**Product length**
**For dsRNA synthesis**		
T7-SPF-L-F	TAATACGACTCACTATAGGG (T_7_)	226 bp
	AGAGGCCTGCTATCAAACGCTAC	
T7-SPF-L-R	TAATACGACTCACTATAGGG (T_7_)	
	AGATCAACCGAGTCAGTGTTCCA	
**For dsRNA synthesis**		
T7-GFP-F	TAATACGACTCACTATAGGG (T_7_)	688 bp
	AAGGGCGAGGAGCTGTTCACCG	
T7-GFP-R	TAATACGACTCACTATAGGG(T_7_)	
	CAGCAGGACCATGTGATCGCGC	
**For real-time PCR**		
Q-SPF-L-F	AAGAGTGACAGACCTGCCAA	189 bp
Q-SPF-L-R	AGGTTCACTGTCCTTGTCACT	
Q-Vg-F	GTGGCTCGTTCAAGGTTATGG	200 bp
Q-Vg-R	GCAATCTCTGGGTGCTGTTG	
Q-VgR-F	AGGCAGCCACACAGATAACCGC	136 bp
Q-VgR-R	AGGCAGCCACACAGATAACCGC	
Actin-F	TGGACTTCGAGCAGGAAATGG	186 bp
Actin-R	ACGTCGCACTTCAGATCGAG	

We generated sense and antisense dsRNAs in separate 20 μL reaction volumes. The dsRNAs were annealed by mixing and incubation at 70°C for 10 min, and then cooling to room temperature over 20 min. Two microliters of RNase A solution (4 mg/mL) and 2 μL RNAase-free DNase (1 U/μL) were added to the reaction tube and incubated in a 37°C water bath for 30 min. The dsRNA was precipitated by adding 110 μL 95% ethanol and 4.4 μL 3 M sodium acetate (pH 5.2), then washed with 0.5 mL 70% ethanol and dried at room temperature. The dried product was dissolved in 50 μL nuclease-free water. The purified dsRNAs were quantified by spectroscopy. The 3rd instar nymphs were treated with dsRNA orally on an artificial diet amended with dsRNA (Fu et al., 2001), with some modifications to the rearing protocol. Previous results indicated that a 0.05 μg/μL dsRNA concentration feeding led to rapid and significant reduction in trehalose phosphate synthase (*NlTPS*) expression level (Chen et al., 2010). We used glass cylinders (15.0 × 2.5 cm diameter) as feeding chambers, with four dsRNA concentrations, 0.0625, 0.06, 0.05, and 0.03 μg/μL. The artificial diet (20 μL) was held between two layers of stretched Parafilm M membrane enclosed at the two open ends of the chamber (the diet capsule). The diet capsule was replaced every second day. The cylinders were covered with a piece of black cotton cloth and the two ends with artificial diet were exposed to light. Insects fed on the diets by puncturing the inner Parafilm M membrane of the diet capsule. Experimental insects were transferred into chambers and maintained on artificial diets for 1 day before initiation of the assays. Twenty 3rd instar individuals were transferred into each chamber, and three chambers were used to create three independent biological replicates. The rearing experiments were carried out in a humidified growth cabinet at 26 ± 2°C, 80% RH and a 16L: 8 D photoperiod; our standard conditions. Mortality was recorded every other day.

### Influence of Dietary dsRNA on Biological Performance Parameters

We determined the effects of dietary dsSPF-L treatments on selected biological performance parameters. We transferred 3rd instar nymphs to capsules containing dsRNA-laced diet. When the nymphs reached fifth (final) instar (10 days), they were collected, individually transferred into a glass jar (12 cm high × 6 cm), and reared on tillering rice plants under standard conditions. One newly emerged, virgin females mated with one newly emerged dsRNA-treated male. One hundred adult females and 200 adult males were collected separately at 2 days after mating for the determination of fresh body weight (*n* = 15, *N* = 3 for mated adult males; *n* = 15, *N* = 3 for mated adult females), soluble ovary protein (*n* = 20, *N* = 3) and soluble fat body protein content (*n* = 20, *N* = 3), juvenile hormone (JH) III titer (*n* = 5, *N* = 3 for mated females or males), ecdysteroid titer (*n* = 5, *N* = 3 for mated females or males), MAGPs content (*n* = 20, *N* = 3), Argine (Arg) content (*n* = 5, *N* = 3) and western blot (*n* = 30, *N* = 3) at 2 days after emergence (DAE). We determined accumulations of mRNAs encoding vitellogenin (Vg; *n* = 5, *N* = 3), and vitellogenin receptor (VgR; *n* = 5, *N* = 3) at 2 DAE. We dissected 15 mated males and 15 mated females. We prepared ovaries (*n* = 15) for immunofluorescence staining analysis. We also transferred individual newly emerged females or males into glass jars (12 cm high × 6 cm), and maintained them separately on tillering rice plants under standard conditions. We collected 100 dsGFP-treated virgin males, 100 dsSPF-L-treated virgin males), and 50 virgin females. We measured the fresh body weight (*n* = 15, *N* = 3 for virgins), JH III, and ecdysteroid titers (*n* = 5, *N* = 3 for virgin females) of each insect. Individual pairs were maintained under standard conditions for oviposition. Two sets (one with dsSPF-L-treated males and one with control males) of 15 copulating pairs were set up to record duration of the pre-oviposition period, oviposition period, adult longevity, and fecundity for each pair. Rice stems were replaced daily during the pre-oviposition period, at 2 day intervals during the oviposition period and 3 day intervals during the female longevity period, until the females died. The number of eggs laid on each rice stem were recoded under a light microscope. We recorded fecundity of 15 mated pairs as the average number of eggs laid/pair. We determined *NlSPF-L* expression patterns in tissues (detailed in the “Results” section) of 20 dsSPF-L treated virgin males (*n* = 15, *N* = 3) at 2 DAE. We used separate virgin males (*n* = 5, *N* = 3) at 1, 3, 5, and 7 DAE to determine *NlSPF-L* silencing efficiency.

### Protein Extraction and Determinations

We prepared soluble proteins from fat bodies and ovaries of 20 mated females using a method similar to Ge et al. (2010b). We isolated individual tissues from adult females at 2 DAE under a zoom streomicroscope (mode XTL20, Beijing Tech Instrument Co., Ltd., Beijing, China) in a cooled petri dish. We isolated tissues into separate, pre-weighed, ice-cold centrifuge tubes and re-weighed the tubes on a Mettler-Toledo electronic balance (EC100 model; 1/10,000 g sensitivity). We added NaCl solution (0.4 M NaCl: 1 M PMSF; v/v at 20 mL NaCl solution/1 g tissue) into the tubes, homogenized the tissues on ice, and centrifuged the tubes at 12,000 × *g* at 4°C for 20 min. We filtered out the upper lipid layer with glass fibers, then added ddH_2_O (1 volume supernatant: 10 ddH_2_O, v/v), and centrifuged again at 4,000 × *g* at 4°C for 20 min. We collected the resulting supernatants and held them at 4°C overnight. We removed the supernatant and dissolved the protein sediment in 1.5 mL of pre-chilled 0.4 M NaCl. We prepared protein samples from male accessory glands (MAGs) of unmated males as reported Ge et al. (2010b) at 2 DAE. We isolated MAGs from 20 males under a zoom-stereomicroscope in a cooled petri dish and placed them in separate, pre-weighted, ice-cold Eppendorf tubes. We added 600 μL of a solution (methanol/distilled water/acetic acid/methyl thioethanol; 80:18:2:0.1; v:v:v:v), homogenized the tissues on ice and centrifuged them at 12,000 × *g* at 4°C for 10 min. After removing the lipid layer, we collected the supernatant. We then added 400 μL of the solution to the sediment in the tubes, centrifuged again, collected the supernatant and combined both supernatants.

We followed Bradford (1976) to measure protein content using Coomassie Brilliant Blue R250 (Shanghai Chemical Agent Co., Ltd., Shanghai, China). We created a standard curve based on a BSA protein standard (Shanghai Biochemistry Research Institute, Shanghai, China). We determined the absorbance at 595 nm in a UV755B spectrometer (Shanghai Precision Instrument Co., Ltd., Shanghai, China) and calculated protein contents according to the standard curve. Protein determinations were repeated three times, with three biologically independent samples.

### Determination of Arg Content and Hormone Titers

We followed the instruction from an insect Arg ELISA kit (Qiandu Biological Technology Co., Ltd., Shanghai, China) to measure Arg content in virgin males at 2 DAE. Each treatment and control were repeated three times (*n* = 5, *N* = 3).

We followed the instructions from an insect double sandwich ELISA kit (Qiandu Biological Technology Co., Ltd., Shanghai, China) for JH III and ecdysteroid titer determinations for virgin males or mated males at 2 DAE. Each treatment and control were replicated three times (*n* = 5, *N* = 3).

### Body Weights and Isolation of IROs

We determined the fresh weights using 15 mated females and, separately, 15 unmated males at 2 DAE. The insects were placed in pre-weighed centrifuge tubes and then weighed using a Mettler-Toledo electronic balance (EC 100 model; 1/10,000 g sensitivity). Each treatment and each control were replicated three times (*n* = 15, *N* = 3).

We isolated IRO (*n* = at least 10 males and, separately, females) from four sets of BPH, control males, experimental males, females mated with control males and females mated with experimental males at 2, 4, and 6 DAE in 1 × phosphate buffered saline (PBS: 137 mM NaCl, 2.68 mM KCl, 1.47 mM KH_2_PO_4_, and 8.10 mM Na_2_HPO_4_, pH 7.0). We fixed the tissues in 3.8% formaldehyde in 1 × PBS for 20 min at room temperature (Ge et al., 2017b), then washed the IROs with 0.2% Triton-X-100 (Sigma, United States) in 1 × PBS three times for 10 min. After washing, we photographed the IROs with a Leica DMR connected to a Fuji FinePixS2 Pro digital camera (Germany).

### qPCR Analysis

We extracted total RNA from five unmated control males (virgin males) at 1, 3, 5, and 7 DAE or untreated mated males and females at 2, 4, at 6 DAE or untreated virgin males and females at 1, 3, and 5 DAE, and from virgin males or virgin females and untreated mated female tissues (*n* = 20, *N* = 3) indicated in Results at 2 DAE using a SV Total Isolation System Kit (model Z3100, Promega Corporation, Madison, WI, United States). We synthesized first-stand cDNA in a 10 μL reaction volume composed of 0.5 μg RNA, 0.5 μL PrimeScript RT enzyme mix I, 0.5 μL Oligo dT primer (50 μM), 2 μL random hexamers (100 μM), 2 μL 5 × PrimeScript Buffer and RNase-free dH_2_O, following instructions of the PrimeScript RT Kit (TakaRa Biotechnology, Dalian, China). The cDNA was reverse transcribed at 37°C for 15 min, 85°C for 5 s and 4°C for 5 min. We extracted and reverse transcribed total RNA from all experimental preparations. All qPCR reactions were performed in triplicate with 96-well plates using a CFX96 touch real-time PCR system (Bio-Rad Co., Ltd., Hercules, CA, United States). Each 10 μL qPCR reaction mixture consisted of 5 μL SYBR mater mix, 0.4 μL of each primer (10 μM), 1 μL of cDNA template equivalent to 50 ng of total RNA and 3.2 μL of deionized water. The qPCR program for *NlSPF-L* was 94°C for 2 min, followed by 40 cycles of 94°C for 5 s, 56°C for 30 s, and 72°C for 30 s. The program for *NlVg* (AB353856) and *NlVgR* (Gu7232977) was 94°C for 1 min, followed by 35 cycles of 94°C for 5 s, 60.4°C (*NlVg*)/60.7°C (*NlVgR*) for 30 s, and 72°C for 30 s. *NlSPF-L* (XM022339255), *NlVg*, and *NlVgR* mRNAs were separately quantified in relation to the stably expressed (Ge et al., 2017a) reference gene, *actin-1* (EU179846). After amplification, a melting curve analysis was performed in triplicate and the results were averaged. The values were calculated using three independent biological samples and the relative *NlSPF-L* transcript level was analyzed by 2^–ΔΔCT^ method (Livak and Schmittgen, 2001). Each treatment and control were replicated three times (*n* = 5, *N* = 3).

### Western Blot Analysis

We conducted SDS-PAGE and immunoblotting as previously described (Ge et al., 2019). In brief, the fat bodies (for NlVg isolation) of treated or control females (*n* = 20) were homogenized using a Tissue Protein Extraction Kit (CWBIO, Taizhou, China). After three freeze/thaw cycles, we centrifuged the homogenates at 12,000 × *g*, 4°C for 30 min and determined protein content in the supernatants following the BCA method (CWBIO, Taizhou, China). We mixed the equivalent protein (30 μg) with 6 × loading buffer for SDS-PAGE (Beyotime, Shanghai, China) and boiled the mixture for 10 min. Following centrifugation at 12,000 × *g* for 10 min, we cooled the samples to room temperature and then loaded them on 8% or 12% SDS-PAGE gels. After separating the proteins, the gels were electro-blotted onto nitrocellulose (NC) membranes (0.45 μm, Solarbio, Beijing, China) using a criterion wet transfer blot apparatus (170471, Bio-Rad, Hercules, CA, United States) at constant 300 mA for 3 h and then washed them with Tris-buffered saline (TBS) for 5 min at room temperature. Blots were blocked at room temperature for 1 h in TBS containing 0.1% Tween 20 (TBST) and 5% (w/v) non-fat powdered milk, followed by washing three times for 5 min each with TBST. Blots were incubated overnight at 4°C with the primary antibody diluted in TBST (1:2000). Blots were then washed three times in TBST for 10 min each and probed with HRP-conjugated goat anti-rabbit IgG (CWBIO, Taizhou, China) as the second antibody in TBST (1:3000). Following extensive rinsing, the immunoreactivity was visualized using ECL reagent (Bio-Rad, Hercules, CA, United States) in the Molecular Imager ChemiDoc XRS System (Bio-Rad). Quantitative analysis of the visible bands in western blot was performed using the ImageJ program^1^ (Schneider et al., 2012). The procedure entails four steps: (a) converting the analyzed file to Grayscale format and subtracting the background with a value of 50; (b) converting the analyzed file to bright band and calculating the density information of a band by hand-drawing an area corresponding to its borders. The information contains area size, mean gray value, minimum and maximum gray value, and integrated density (IntDen); (c) extracting the IntDen values of all the measured bands; (d) normalization by dividing the Vg IntDen values against the β-actin values. We obtained the primary antibody, anti-β-actin, from Abcam (Cambridge, United Kingdom), and anti-rabbit NlVg was generously gifted by Prof. Haijun Xu (Zhejiang University).

### Immunofluorescence Microscopy

We adapted the protocol of Zhuo et al. (2018) for NlVg antibody staining. We isolated ovarioles from ovaries of mated females at 2 DAE in cold PBS (pH 7.4). After washing three times in PBS, we fixed the tissues in 4% paraformaldehyde for 2 h at room temperature and washed them in PBST containing 0.1% Triton X-100 three times for 30 min each. We blocked the specimens in PBST containing 5% normal goat serum for 4 h at room temperature, followed by incubation with anti-NlVg (1:500, gifted by Prof. Haijun Xu, Zhejiang University) in PBST containing 2% normal goat serum and 3% BSA overnight at 4°C. After rinsing with PBS 3X, for 5 min each, under dim light, we added Alexa Fluor 488-labeled goat anti-rabbit secondary antibody (1:500) (Beyotime, Shanghai, China) in PBST containing 2% goat serum and 3% BSA. We incubated the samples at room temperature for 1 h and counterstained the nuclei with 100 nM 4’,6-diamidino-2-phenylindole (DAPI) (Beyotime, Shanghai, China) in PBST for 10 min at room temperature. We mounted the specimens on glass slides and washed again with PBS 3X, 5 min each and viewed them under a Leica TCS SP5 confocal microscope (Leica Microsystems, Solms, Germany). We observed DAPI (blue) and NlVg (green) fluorescence at 405 and 488 nm, respectively, with gain and offset set to 1% and the high voltage set to 400–600. We used a line sequential scanning mode for image capture at a 1,024 × 1,024 pixel resolution.

### Phylogenetic Analyses

We constructed a phylogenetic tree of SPF-L homologs using the Maximum Likelihood method with MEGA6 software (Tamura et al., 2011; Lu et al., 2016). Phylogenetic analysis of the SPF-L amino sequences comes from BPH and other insects. BPH and other insect SPF-L amino sequences were used as a query to search for homologs in the NCBI mRNA and EST library. All of the homologs were then aligned in a multiple sequences alignment using CLUSTAL X (Jeanmougin et al., 1998) and edited with GeneDoc software.

### Population Growth

We carried out a population growth experiment following Ge et al. (2017b), using two treatment groups: females mated with control males and females mated with experimental males. We arranged the experiment in a randomized complete block design with five replicates. Four pairs of newly emerged BPHs were released onto rice plants at the tillering stage and covered with a nylon cylindrical cage (20 cm diameter × 80 cm height, screen size: 80-mesh) in each pot. When neonates of the next generation emerged, we checked each treatment group daily and counted the neonates. We transferred them into new plastic plots as just described until the original females died. We examined the neonates every 2 days until adult emergence and recorded numbers of females and males. We counted all adults from the next generation, recorded the numbers of unhatched eggs on the rice sheaths on which the adults had been feeding and recorded hatch rates as all adults/all adults plus unhatched eggs. A population growth index (PGI) is expressed by the ratio of *N*1/*N*0, where *N*1 is the total number of adults from the next generation divided by *N*0, the number of adults released (*N*0 = 8).

### Statistics Analyses

**FIGURE 1:**
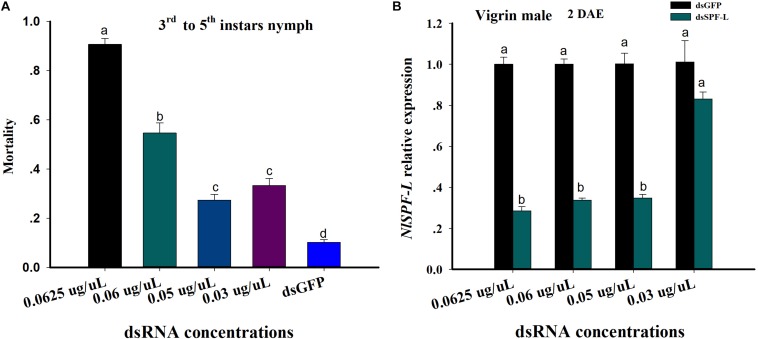
dsRNA treatments influence nymphal BPH mortality and accumulation of mRNAs encoding NlSPF-L. **(A)** The histogram bars show mean BPH male and nymph mortality as proportions. The error bars indicate one SEM, *n* = 50, *N* = 3 independent biological replicates. **(B)** The histogram bars show mean silencing efficiency of *NlSPF-L* in dsRNA-treated males at 2 DAE, *n* = 5, *N* = 3 independent biological replicates (Student’s *t*-test). The histogram bars with different letters are significant difference at *p* < 0.05.

Normality and homogeneity data of variance was examined by the Shapiro–Wilk test and Bartlett test, which showed that no transformations were needed. RNAi silencing efficiency of *SPF-L* at different DAE (Figures 1B, 4H) was performed with using Student’s t-test. A one-way ANOVA followed by Tukey’s honestly significant difference (HSD) multiple comparison test was performed to analyze other data. All analyses were performed with the data procession system (DPS) of Tang and Feng (2002). Significant differences were considered at *P* < 0.05. All values are expressed as mean ± SEM. The relative gray values for western blot analysis were determined in the NIH ImageJ software (see text footnote 1).

## Results

### Dietary dsSPF-L Treatments Influenced Mortality

From the 3rd instar to 5th instar nymph, mortality of nymph BPH increased with increasing dietary dsSPF-L concentrations compared to dsGFP treatment control (Figure 1A; *F* = 186.3, df = 4, 14, *P* < 0.001). Mortality of nymph BPH feeding 0.05 and 0.03 μg/μL dsSPF-L concentrations were not significantly difference (Figure 1A). The silencing efficiencies of high three dietary dsRNA concentrations were similar at approximately 70% compared to dsGFP treatments controls (Figure 1B; *F* = 105.4, df = 3, 23, *P* < 0.001) and the lowest dose was not different from controls (*F* = 2.7, df = 1, 8, *P* = 0.1769). We selected the 0.05 μg/μL dsRNA concentration for all experiments based on mortality and silencing efficiency of *NlSPF-L* in virgin BPH males at 2 DAE.

### Phylogenetic and Sequence Analyses of the BPH SPF-L

**FIGURE 2:**
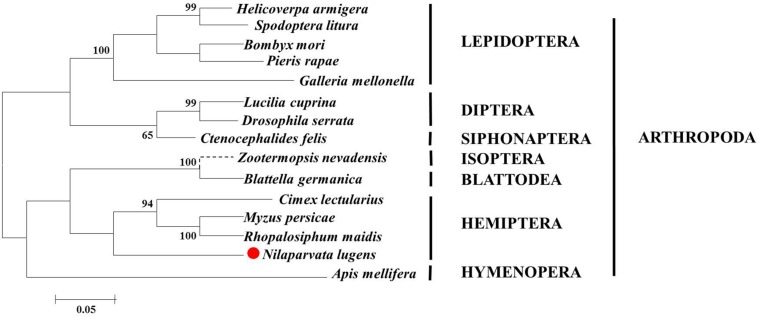
Phylogenetic analysis, based on amino acid sequences, of the SPF-L sequences from BPH and other insects. Sequences were retrieved from GenBank protein database, including *Helicoverpa armigera* (XP021184618), *Zootermopsis nevadensis* (XP021941207), *C. lectularius* (XP014242913), *Blattella germanica* (PSN58039), *Bombyx mori* (XP004927858), *Spodoptera litura* (XP022829415), *Pieris rapae* (XP022120009), *Myzus persicae* (XP022167433), *Rhopalosiphum maidis* (XP026813571), *Apis mellifera* (XP394140), *Lucilia cuprina* (XP023302715), *Ctenocephalides felis* (XP026461767), *Drosophila serrata* (XP020809590), and *Nilaparvata lugens* (XP022194947). The phylogentic tree of SPF-L homologs was constructed using the Maximum Likelihood method with MEGA6 software. Bootstrap values are shown in the nodes. Branch lengths are proportional to sequence divergence. The scale bar indicates the average number of amino acid substitutions per site.

**FIGURE 3:**
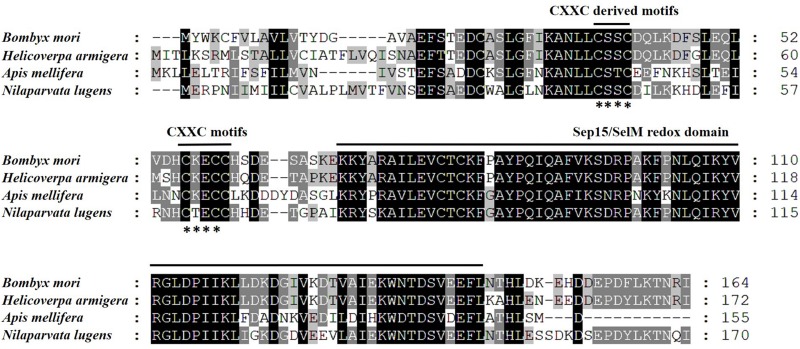
Amino acid sequence alignment of SPFs of *B. mori*, *H. armigera*, *A. mellifera*, and *N. lugens* using CLUSTAL X. Letters shaded in black and gray indicate the identical and similar amino acid residues, respectively; asterisk (^∗^) indicates conserved CXXC-motifs or CXXC-derived motifs.

We opted to do these amino acid sequences since online blast results showed the homology of them was all beyond 60% against the target one. Thus, some sequences with less similarity are not displayed. Phylogenetic analysis based on amino acid sequences indicates that selenoproteins from the species listed in Figure 2 clustered reasonably close together. In particular, the four hemipteran proteins were quite close, like *Myzus persicae*, *Rhopalosiphum maidis*, and *Cimex lectularius*, suggesting the SPF-Ls were evolutionarily conserved. Multiple sequences alignment of *B. mori*, *H. armigera*, *A. melllifera*, *N. lugnes* showed conserved residues primarily from Sepl15/SelM redox domain as a thiroredoxin-like and active-site redox motif, and have selenocystine-containing CXXC motifs and CXXC derived motifs as active-residues (Figure 3).

### *NlSPF-L* Expression Characteristic and Silencing Efficiency Analysis

**FIGURE 4:**
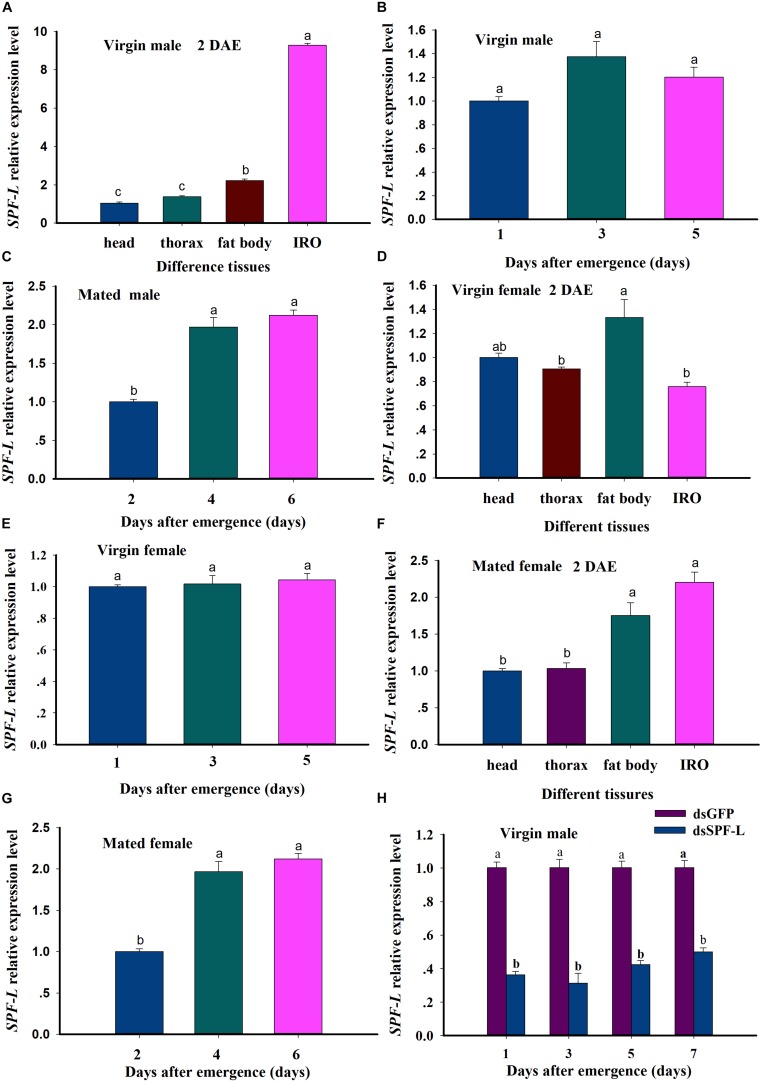
Relative accumulations of mRNA encoding the BPH SPF-L. **(A)** The histogram bars indicate accumulations of mRNA in the indicated body segments and tissues of virgin male at 2 DAE. **(B,C)** The histogram bars indicate the mean *SPF-L* expression level in virgin males and mated males (*n* = 5, *N* = 3). **(D,E)** The histogram bars indicate the mean *SPF-L* expression level in the tissues of virgin female (*n* = 20, *N* = 3) and at 2, 4, 6 DAE in virgin female (*n* = 5, *N* = 3). **(F,G)** The histogram bars indicate the mean SPF-L expression level in the tissues of mated female (*n* = 20, *N* = 3) and at 2, 4, 6 DAE in mated female (*n* = 5, *N* = 3). The error bars indicate one SEM. The mRNA expression value of adult female head was converted to 1. **(H)** The histogram bars show mean relative mRNA accumulation (*n* = 5, *N* = 3 independent biological replicates) at 1, 3, 5, and 7 DAE in virgin males and the error bars represent one SEM. Gene expression was normalized to the β-actin reference gene. The histogram bars with different letters were significant difference at *p* < 0.05 (Student’s *t*-test).

We recorded relative accumulations of mRNA encoding SPF-L. Thoraces produced the lowest amounts, fat bodies slightly more and IRO much higher levels, up approximately 7.9-fold compared to heads in virgin male at 2 DAE (Figure 4A; *F* = 2,319.9, df = 3, 11, *P* < 0.001). *SPF-L* mRNA expression level in virgin males was no significant difference at 1, 3, and 5 DAE (Figure 4B; *F* = 4.3, df = 2, 8, *P* = 0.0684), and in mated males at 4 and 6 DAE was higher than that of mated males at 2 DAE (Figure 4C; *F* = 53.2, df = 2, 8, *P* = 0.0002). Meanwhile, we found *SPF-L* expression level in fat body of virgin females was higher than that of other tissues at 2 DAE (Figure 4D; *F* = 9.6, df = 3, 11, *P* = 0.0049), and no significant difference in virgin females at 1, 3, and 5 DAE (Figure 4E; *F* = 0.29, df = 2, 8, *P* = 0.7594), SPF-L expression level of fat body and IRO in mated females at 2 DAE was aslo higher than those of head and thorax in mated females (Figure 4F; *F* = 24.1, df = 3, 11, *P* = 0.0002), in mated females at 4 and 6 DAE was higher than that of mated females at 2 DAE (Figure 4G; *F* = 547.6, df = 2, 8, *P* < 0.001).

These results indicated *SPF-L* mRNA specifically expressed in fat body and IRO of virgin males, and high expression in fat body of virgin females. *SPF-L* mRNA expression levels was relatively stable in either the virgin females or the virgin males, *SPF-L* expression level was markedly increased in either mated females or mated males, suggesting SPF-L transferred to female via copulation. dsSPF-L treatments led to reduced (*F* = 500.1, df = 1, 23, *P* < 0.001) accumulations of mRNAs encoding SPF-L compared to dsGFP treatments at 1, 3, 5, and 7 DAE (Figure 4H), although the amounts of reduction were similar at all 4 days (*F* = 2.1, df = 3, 23, *P* = 0.1367). Also, no significant interactions between RNAi and DAE were observed (*F* = 2.2, df = 3, 23, *P* = 0.1328) (Figure 4H).

### Influence of Silencing *SPF-L* on Males

**FIGURE 5:**
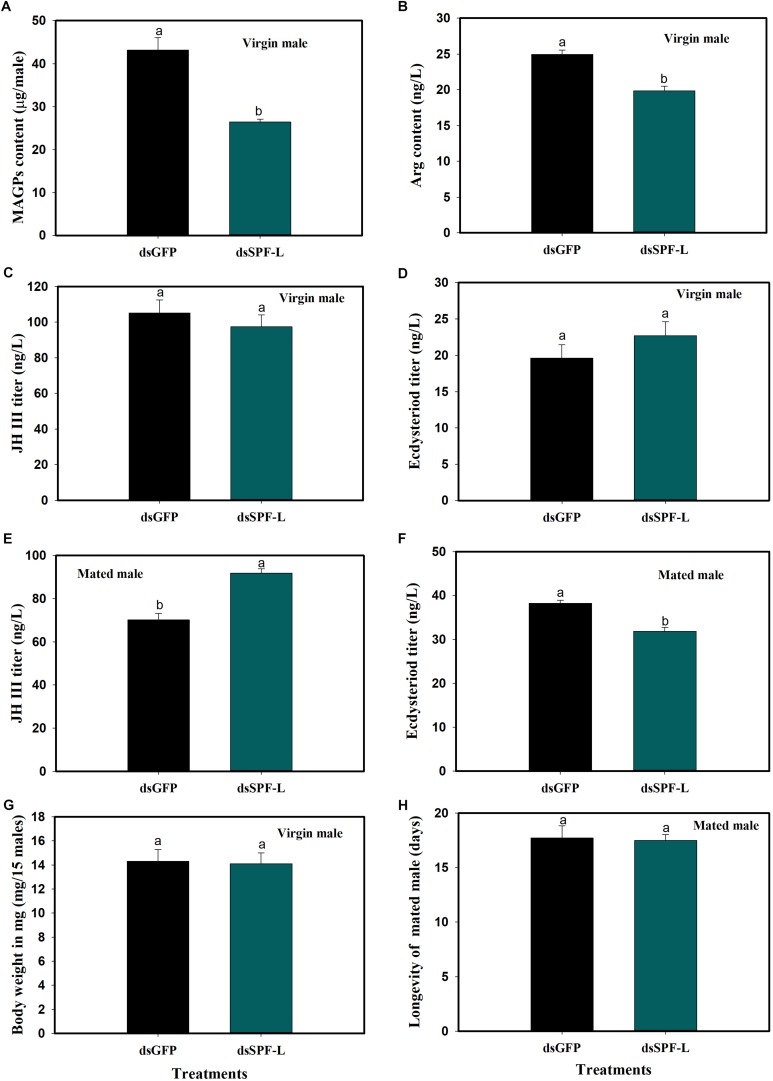
Effects of dsSPF-L treatments on male BPH parameters. **(A)** The histogram bars show mean of MAGs content in dsSPF-L-treated virgin males at 2 DAE (*n* = 20, *N* = 3 independent biological replicates). **(B)** The histogram bars show mean of Arg content in dsSPF-L-treatedvirgin males at 2 DAE (*n* = 5, *N* = 3 independent biological replicates). **(C)** The histogram bars show mean JH III titer in dsSPF-L-treated virgin male at 2 DAE (*n* = 5, *N* = 3 independent biological replicates). **(D)** The histogram bars show mean ecdysteriod titer in dsSPF-L-treated virgin male at 2 DAE (*n* = 5, *N* = 3 independent biological replicates). **(E)** The histogram bars show mean JH III titer in dsSPF-L-treated mated males at 2 DAE (*n* = 5, *N* = 3 independent biological replicates). **(F)** The histogram bars show mean of ecdysteroids titer in dsSPF-L-treated mated males at 2 DAE (*n* = 3 independent biological replicates). **(G)** The histogram bars show mean body weight in dsSPF-L-treated virgin males at 2 DAE (*n* = 15, *N* = 3 independent biological replicates). **(H)** The histogram bars show mean longevity in dsSPF-L-treated males (*n* = 15 independent biological replicates). All error bars indicate 1 SEM. The histogram bars with different letters were significant difference at *p* < 0.05 (Student’s *t*-test).

In virgin males, dietary dsSPF-L treatments led to reduced MAGP content (*F* = 421.2, df = 1, 5, *P* = 0.0001), down by 39% compared to dsGFP treatments (Figure 5A) and to reduced Arg content at 2 DAE (*F* = 32.4, df = 1, 5, *P* = 0.0047), down by 20% (Figure 5B). The dsRNA treatments did not influence JH III (Figure 5C; *F* = 0.6, df = 1, 5, *P* = 0.481) or ecdysteroid titers (Figure 5D; *F* = 1.4, df = 1, 5, *P* = 0.3082), nor body weights (Figure 5G; *F* = 0.03, df = 1, 5, *P* = 0.8703). In mated males, the dsRNA treatments led to increased JH III titers (Figure 5E; *F* = 35.2, df = 1, 5, *P* = 0.004) and decreased ecdysteroid titers (Figure 5F; *F* = 29.5, df = 1, 5, *P* = 0.0056) at 2 DAE. The treatments did not influence longevity of mated males (Figure 5H; *F* = 0.046, df = 1, 29, *P* = 0.8317).

### dsSPF-L Treated Males Influence Their Mating Partners

**FIGURE 6:**
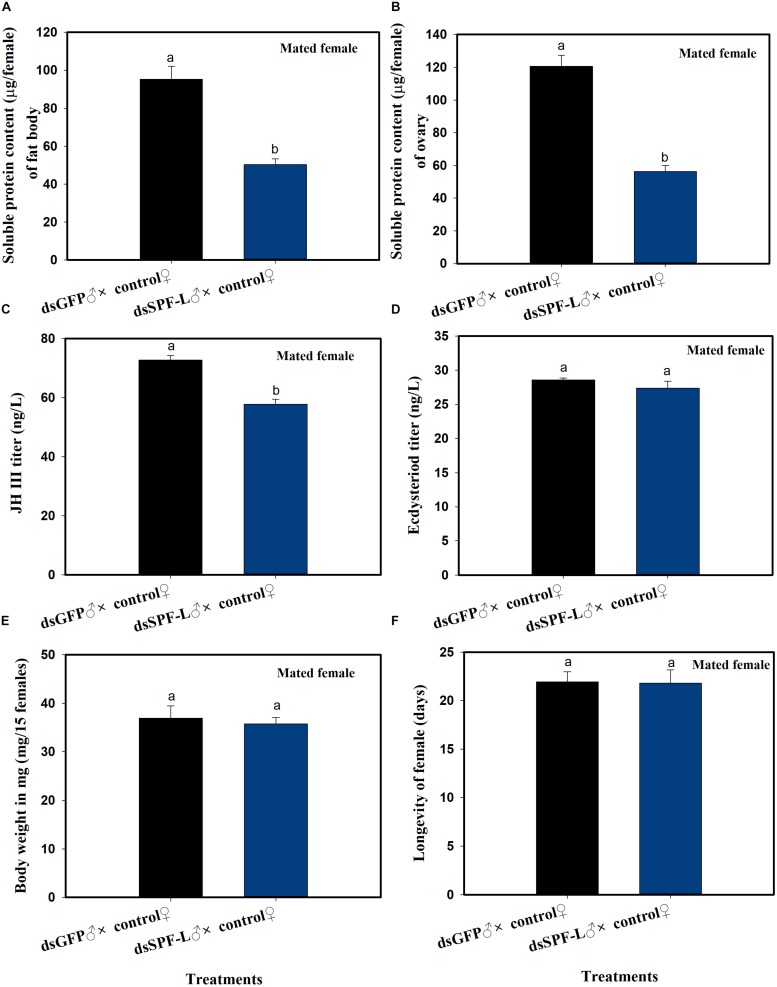
The influence of mating with dsSPF-Ltreated males on females at 2 DAE. **(A)** The histogram bars show mean fat body soluble protein content in mated females (*n* = 20, *N* = 3 independent biological replicates). **(B)** The histogram bars show mean ovarian soluble protein content in mated females (*n* = 20, *N* = 3 independent biological replicates). **(C)** The histogram bars show mean JH III titer (*n* = 3 independent biological replicates). **(D)** The histogram bars show mean ecdysteriod titer (*n* = 3 independent biological replicated). **(E)** The histogram bars show mean body weight (*n* = 15, *N* = 3 independent biological replicated). **(F)** The histogram bars show mean longevity of females (*n* = 15 independent biological replicated). All error bars indicate 1 SEM. The histogram bars with different letters were significant difference at *p* < 0.05 (Student’s *t*-test).

Mating with experimental males led to reduced fat body (*F* = 36.4, df = 1, 5, *P* = 0.0038) and ovarian (*F* = 71.6, df = 1, 5, *P* = 0.0011) protein contents at 2 DAE, as well as to reduced JH III titers (Figure 6C; *F* = 45.3, df = 1, 5, *P* = 0.0025) in their partners (Figures 6A–C), compared to controls at 2 DAE. Similar experimental matings did not influence ecdysteroid titers (Figure 6D; *F* = 1.3, df = 1, 5, *P* = 0.3175), body weights (Figure 6E; *F* = 0.169, df = 1, 5, *P* = 0.7023) at 2 DAE, nor longevity (Figure 6F; *F* = 0.006, df = 1, 29, *P* = 0.9388) in partners.

### dsSPF-L-Treated Males Influence *NlVg* Gene Expression and Vg Synthesis

**FIGURE 7:**
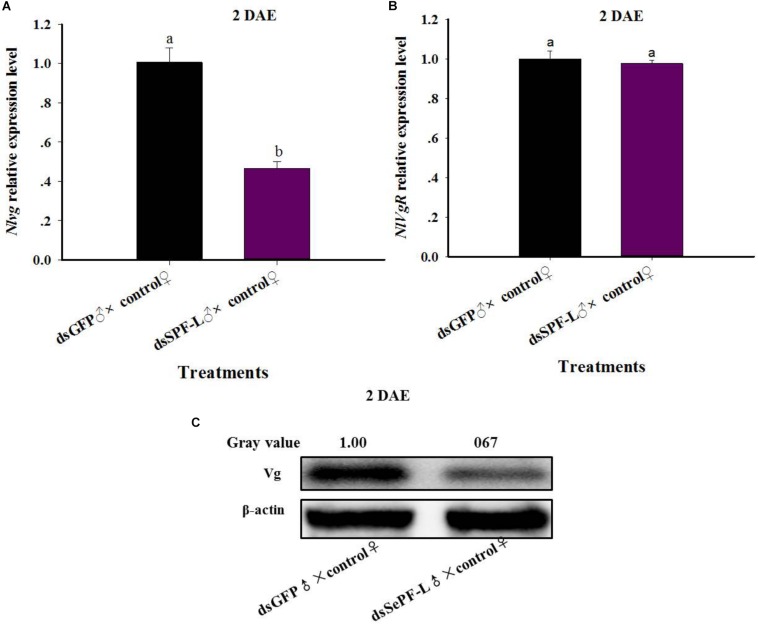
The influence of mating with dsSPF-Ltreatedmales on accumulations of mRNAs encoding Vg, VgR, and Vg protein synthesis. **(A)** The histogram bars show mean *NlVg* expression level at 2 DAE (*n* = 5, *N* = 3). **(B)** The histogram bars show mean NlVgR expression level at 2 DAE (*n* = 5, *N* = 3). **(C)** Western blot analysis of mating with dsSPF-L-treated males led to reduced fat body Vg protein levels of Vg using antibody-rabbit Vg in at 2 DAE (*n* = 5, *N* = 3). β-actin antibody was used as the loading control. The relative gray values normalized to β-actin were marked above corresponding bands. The relative gray values of dsGFP treatment was looked on as 1. The histogram bars with different letters were significant difference at *p* < 0.05 (Student’s *t*-test).

**FIGURE 8:**
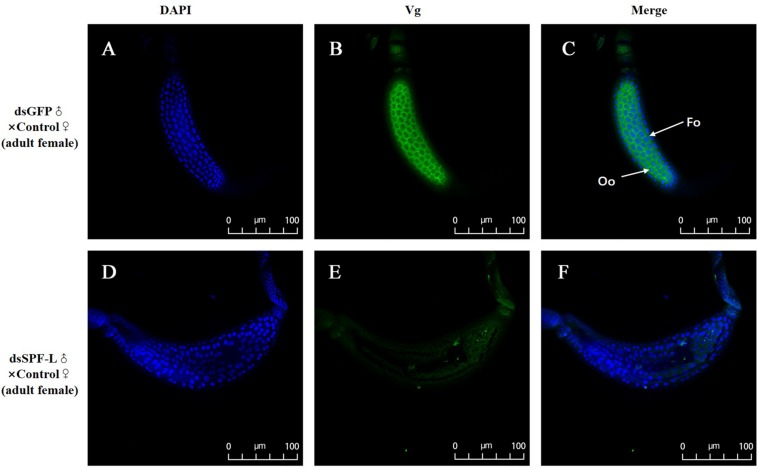
Influence of mating with dsSPF-L-treated males on BPH ovarian Vg synthesis at 2 DAE. **(A,D)** DAPI stained nuclei (blue). **(B,E)** Vg protein detected with a goat anti-rabbit IgG labeled with Dylight 488 (green). **(C,F)** Merged images. The fluorescence staining images were captured using a Zeiss LSM 780 confocal microscope. In panel **(C)**, Fc: follicle cells; Oo: oocytes.

Mated with dsSPF-L-treated males led to reduced accumulations of mRNAs encoding NlVg (Figure 7A; *F* = 46.9, df = 1, 5, *P* = 0.0024), but not NlVgR (Figure 7B; *F* = 0.285, df = 1, 5, *P* = 0.6218), in their partners, down by 354% compared to controls at 2 DAE. We confirmed the reduced *NlVg* expression by western blot analysis (Figure 7C; Gray value = Vg/β-actin = 0.77). Fluorescence staining showed that the uptake of Vg by the follicle cells was impeded in females that mated with dsSPF-L-treated males, but not after mating with control males (Figure 8).

### Influence of dsSPF-L-Treated-Males on Female Reproduction

**FIGURE 9:**
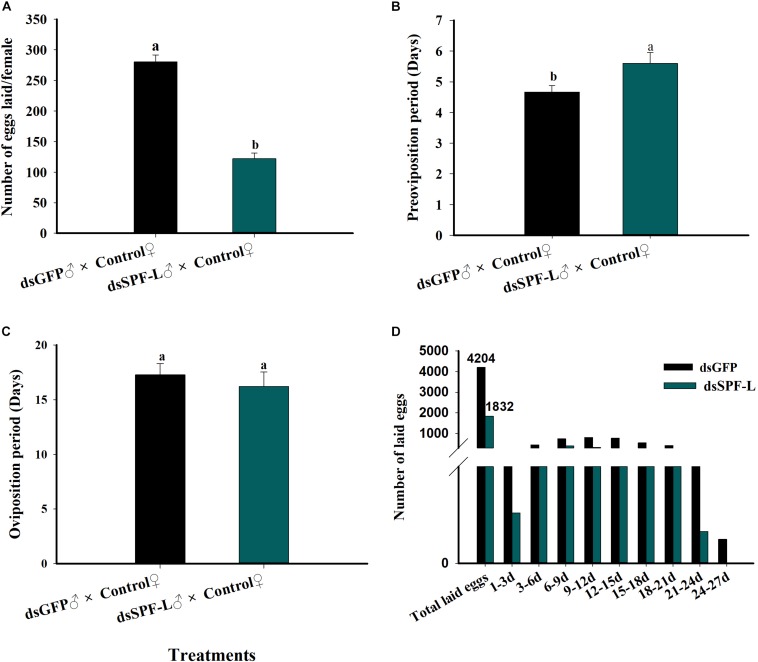
Influence of mating with dsSPF-L treated males on female reproductive parameters. **(A)** The histogram bars show mean numbers of eggs deposited (*n* = 15 independent biological replicates). **(B)** The histogram bars show duration of the pre-oviposition period (days) (*n* = 15 independent biological replicates). **(C)** The histogram bars show the duration of the oviposition period (days) (*n* = 15 independent biological replicates). **(D)** The histogram bars show numbers of eggs deposited during the indicated oviposition intervals in days. *n* = 15 mating pairs. The histogram bars with different letters are significant difference at *p* < 0.05 (Student’s *t*-test).

Mating with dsSPF-L-treated males led to reduced numbers of laid eggs per female (*F* = 115.5, df = 1, 29, *P* < 0.001), down by 56% compared to controls (Figure 9A); elongated pre-oviposition periods, up by 20% (Figure 9B; *F* = 5.2, df = 1, 29, *P* = 0.0299), and no change in the oviposition period (Figure 9C; *F* = 0.4, df = 1, 29, *P* = 0.5322). Figure 9D shows total numbers of deposited eggs following experimental matings (1,832 eggs) and control matings (4,204). The influence of experimental matings was significantly lower compared to control matings during two intervals, 1–3 d and 21–24 d. For the 24–27 d interval, control matings led to very few deposited eggs and egg laying was abolished after experimental matings (Figure 9D).

### dsSPF-L Treatments Led to Disrupted IRO Development in Males and Their Partners

**FIGURE 10:**
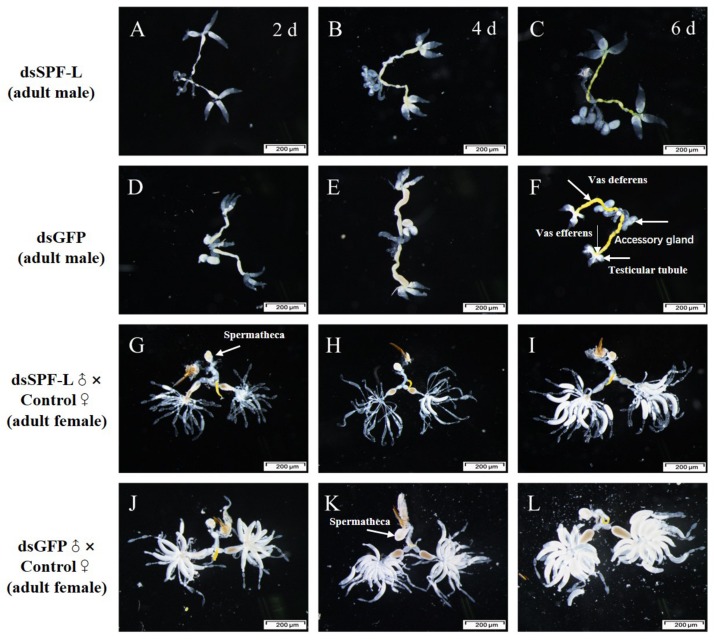
Influence of dsSPF-L-treated male on the IROs of males and their mating partners at 2, 4, and 6 DAE, *n* ≥ 15. DAE are indicated in upper right corners of panels **(A–C)**. **(A–C)** IROs from treated males, just over panels **(D–F)**, with images of IROs from control males. **(G–I)** IROs from partners after mating with dsSPF-L-treated males, just atop panels **(J–L)** with images of IROs from partners of control males.

Sperm are produced in testes, then pass out of the testes through ducts called vasa efferentia and collect in seminal vesicles. Similar ducts, vasa deferentia, transfer sperm from seminal vesicles into an ejaculatory duct. Males also feature accessory glands in which seminal fluids are produced. Some MAGs also produce spermatophores, in which sperm and seminal fluids are transferred to mating partners (Figure 10F). We found that dsSPF-L treatments led to disrupted IRO development in males, seen as transparent testicular tubules and vas efferent, while vas deferens became thin and light-colored (Figures 10A–C) compared to controls at 2, 4, and 6 DAE (Figures 10D–E). After mating with dsSPF-L-treated males, the ovarioles contained far fewer ripe banana-shaped oocytes (Figures 10G–I) compared to controls (Figures 10J–L) at 2, 4, and 6 DAE. We recorded retarded ovarian development with fewer mature eggs in mating partners of dsSPF-L-treated males (Figures 10G–I) over the same time-frame. These females absorbed less Vg into follicle cells which impeded oocytes and ovarian development (Figures 10G–I).

**FIGURE 11:**
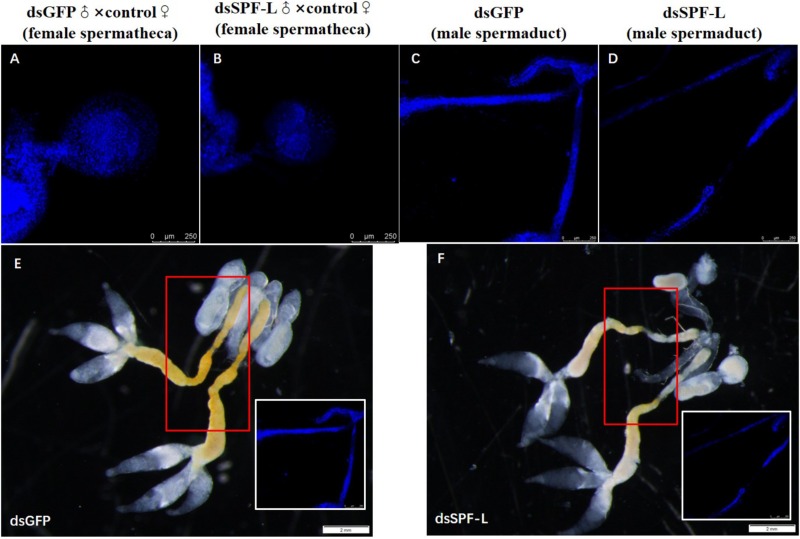
The influence of dsSPF-L-treated males on morphology of male spermaducts and spermathecae of their partners, with DAPI staining at 2 DAE. **(A)** An expanded spemateca filled with sperm and seminal fluids. **(B)** A much smaller, unfilled spermatheca after mating with a dsSPF-L-treated partner. **(C)** An intact spermaduct from a control-treated BPH. **(D)** A much reduced spermaduct from a SPF-L-treated BPH. **(E,F)** Display the overall male reproductive anatomy of dsSPF-L-treated and dsGFP control BPHs. The images were captured using a Zeiss LSM 780 confocal microscope.

Matings with dsSPF-L-treated males led to very small spermathecae compared to spermathecae from controls (Figures 11A–B). This may be due to the absence of transferred sperm because the vas deferens is very much smaller in experimental, compared to control, males (Figures 11C–D). The smaller vas deferens may not support sperm transfer. Figures 11E–F shows the anatomical locations of the spermaducts shown in Figures 11C–D.

### Matings With dsSPF-L-Treated Males Led to Reduced Offspring, Hatching Rate, Gender Ratio, and Population Index

Matings with dsSPF-L-treated males led to reduced numbers of offspring (Table 2; *F* = 78.7, df = 1, 9, *P* < 0.001), down by 43% compared to controls, hatch rates (*F* = 81.2, df = 1, 9, *P* < 0.001), down by 28% and PGI, without influencing the gender ratio of offspring.

**TABLE 2:** Mating with dsSPF-L-treated males led to reduced egg laying, hatch rates, and PGI.

**Treatments**	**Number of offspring**	**Hatching rate**	**Gender ratio**	**PGI(*N*1/*N*0)**
dsGFP♂ × Control♀	782.4 ± 35.3a	0.89 ± 0.01a	1.11 ± 0.03a	97.8
dsSep♂ × Control♀	442.8 ± 14.7b	0.64 ± 0.03b	1.10 ± 0.04a	55.4

*The data in the table are means ± SEM (*N* = 5). Different lowercase letters indicate significant difference between dsGFP-treated control group and RNAi-silenced group at *P* < 0.05 by Student’s *t*-test. *N*0 = 4 pairs (female × male).*

## Discussion

The data presented in this paper form lines of evidence supporting our hypothesis that male SPF-L influences female fitness, seen as increased offspring. Several points make up the argument. First, phylogenetic analysis shows the BPH SPF-L clusters most closely with two other hemipteran SPF-Ls at the amino acid level. Second, the gene is expressed in several tissues, most highly in the IRO of virgin males. Third, silencing the gene with a dietary dsRNA construct led to reduced MAGP and reduced Arg in unmated males and influenced JH III and ecdysteroid titers in mated males. Fourth, silencing *NlSPF-L* in males led to reduced fat body and ovarian protein contents and reduced JH III titers in their mating partners. Fifth, mating with dsSPF-L-treated males led to substantial reductions in *Nlvg* expression and reductions in Vg protein in females. Sixth, mating with dsSPF-L-treated males led to reduced female reproductive fitness, registered as reduced oviposition, reduced offspring numbers, reduced egg hatch rates and reduced PGI values. Finally, dsSPF-L treatments led to serious disruptions in IRO development in males and in their mating partners. We infer our hypothesis is potently supported.

SPF acts in oxidative stress, endoplasmic reticulum stress and carcinogenesis (Ferguson et al., 2006). It is conserved across animal phyla, and we understand that genes in *Caenorhabditis elegans*, *Brugia malayi*, and *Drosophila melanogaster* encode homologous proteins that contain cysteine in place of selenocysteine (Gladyshev et al., 1998). The dendrogram protein highlights a consecutive 75 amino acid segment (75-149) homologous to other hemipteran species, *M. persicae* and *R. maidis.* We infer that SPFs are relatively conserved in evolutionary processes. The protein sequence alignment of the BPH SPF-L also shows conserved residues primarily from Sep15/Se1M redox domain (amino acids 75-149) as a thioredoxin-like and active-site redox motif, harboring selenocysteine-containing CXXC-like motifs (amino acids 61-64) and CXXC-derived motifs (amino acids 43-46) as active-site cysteine residues. We infer from these amino acid features that the BPH SPF-L also acts in oxidative stress.

Insect seminal fluid proteins are important for fertilization and for males in sexual competition, such as manipulating post-mating physiology and behavior in females (LaFlamme and Wolfner, 2013). The current results indicated that SPF-L mRNA was expressed in males, unmated females, and mated females. SPF-L expression level significantly increased in mated males and female, suggesting SPF-L mRNA level was induced by copulation. Yu et al. (2016) reported that SPF-L was found in the male reproductive tract and the female reproductive tract, and was transferred to females after mating. Selenoproteins in human seminal fluid are likely important for protecting during storage (Michaelis et al., 2014). Silencing the BPH *SPF-L* in males led to reduced MAGPs contents. MAGPs help ensure male paternity via several mechanisms, such as sperm mobility, sperm storage, stimulation of ovulation/oviposition, and egg protection (Gillott, 2003). Two examples of this are the example of the, cricket, *Teleogryllus commodus*, mating system, males transfer compounds that release egg-laying behavior in newly mated females (Loher et al., 1981). This promotes egg fertilization before female partners can re-mate with other males. Similarly, Garcia-Gonzalez and Simmons (2005) reported that sperm viability is positively correlated with paternity assurance in the related cricket, *Teleogryllus oceanicus* (Leguillou). Thus, seminal fluids contribute to male and, separately, female fitness, here taken as reproductive success and long-term persistence of genes.

Triazophs exposure enhances BPH reproduction through several mechanisms, one of which is recorded as increased MAGPs transfer to females (Ge et al., 2010a; Wang et al., 2010). *NlSPF-L* silencing also led to reduced transfer of Arg, a nitric oxide (NO) precursor. NO promotes spermatogenesis and enhances fertilization (Zhang and Zheng, 1996; Fujisawa et al., 2001). We infer that dietary dsSPF-L treatments influence spermatogenesis and sperm motility. The images of spermathecal staining indicate reduced sperm transfer from dsSPF-L-treated males. Our interpretation is that the dsSPF-L treatments led to reduced transfer of MAGPs content, Arg content and number of sperm, which translates into reduced egg production and deposition.

Seminal fluids have complex compositions, including multiple inorganic ions, amino acids, carbohydrates, lipids, proteins (sex peptides) and hormones, such as JH or ecdysteroids (Wolfner, 1997; Park et al., 1998). Insect seminal fluid proteins and peptides are transferred to action sites within and outside the female reproductive tract. The fluids lead to numerous responses in females (Soller et al., 1997; Chapman et al., 2003; Liu and Kubli, 2003; Clifton et al., 2014). Silencing *NlSPF-L* in unmated males did not affect JH III titers compared to controls, although the outcome was otherwise in mated males, with increased JH III titer and reduced JH III in mated females. We inferred that silencing *NlSPF-L* might influence the mating frequency or times of males, resulting in lower sperm, JH III, and other transmitted nutritional substances, which may be partially attributed to the deformed and thin vas efferens. *Hyalophora cecropia* and *Heliothis virescens* transfer JH to females within seminal fluid, which results in increased JH titers in female hemolymph and promotes egg development as well as maturity (Wolfner, 1997; Park et al., 1998). Furthermore, we found silencing *SPF-L* led to down-regulation of *NlVg* expression, and did not influence *NlVgR* expression at 2 DAE. Although *NlVgR* expression level was not influenced by dsSPF-L treatments, suggesting *NlVg* and *NlVgR* expression time might not reach a consensus. Tufail et al. (2010) reported Vg expression was regulated by JH in *N. lugens*, and silencing *NlVgR* significantly inhibited ovarian development and accumulation of the Vg protein in the hemolymp (Tufail et al., 2010), and the mRNA and protein levels of *NlVg* were up-regulated by JHIII (Lu et al., 2015). These evidences indicated that RNAi-SPF-L indirectly influenced JH III titer, resulting in down-regulation of *Vg* expression and Vg protein synthesis, and inhibiting ovarian development and fecundity. The previous results demonstrated that MAGPs of TZP-treated males significantly increased compared to untreated controls along with JH III titer (Ge et al., 2010b; Wang et al., 2010), thus stimulating female fecundity via mating (Wang et al., 2010). Zhuo et al. (2018) Silencing the BPH doublesex gene, *Nldsx*^*M*^, resulted in pseudofemales, with malformed sexual anatomy that led to male infertility. Here, by nuclear staining analysis of female spermatheca we found that mating with dsSPF-L-treated males led to reduced spermathecal sizes, probably due to lack of sperm. The present study provided the valuable information that SPF-L has potential as a novel target gene for control of phloem-feeding pest insects. In *D. melanogaster*, selenoprotein biosynthesis was activated by the Ras/MAPK signaling pathway by the redox (Morey et al., 2001). However, how SPF-L signaling pathway regulates reproduction in *N. lugens* needs further research.

## Data Availability Statement

All datasets generated for this study are included in the manuscript/supplementary files.

## Author Contributions

LG designed the research. YZ, HG, QW, ZZ, and SZ conducted the experiments. LG wrote the first draft of the manuscript. QS and DS revised the final draft of the manuscript.

## Conflict of Interest

The authors declare that the research was conducted in the absence of any commercial or financial relationships that could be construed as a potential conflict of interest.

## Footnotes

1 https://imagej.nih.gov/ij/
